# Linkage to Outpatient Methadone Treatment From the Emergency Department and Hospital

**DOI:** 10.1111/acem.70135

**Published:** 2025-08-22

**Authors:** Alice Zhang, Jasmine Barnes, James Sherman, Nicole O'Donnell, Rosemary Velez, Samantha Huo, Ashish Thakrar, Margaret Lowenstein, Jeanmarie Perrone, Austin S. Kilaru

**Affiliations:** ^1^ Department of Emergency Medicine, Perelman School of Medicine University of Pennsylvania Philadelphia Pennsylvania USA; ^2^ Center for Addiction Medicine and Policy, Penn Medicine Philadelphia Pennsylvania USA; ^3^ Merakey Parkside Recovery Philadelphia Pennsylvania USA; ^4^ Department of General Internal Medicine, Perelman School of Medicine University of Pennsylvania Philadelphia Pennsylvania USA; ^5^ Leonard Davis Institute of Health Economics University of Pennsylvania Philadelphia Pennsylvania USA

As morbidity and mortality from opioid use disorder (OUD) remain high, a priority has been to link emergency department (ED) patients to treatment [1]. The most effective strategy is to initiate medication, typically buprenorphine, with navigation to timely outpatient follow‐up care [1, 2]. Although national efforts have succeeded in changing clinical guidelines, removing policy barriers, and building scalable linkage programs, treatment rates after ED and hospital care remain low [1].

Methadone has comparable effectiveness to buprenorphine for the treatment of OUD [1]. However, ED linkage programs do not typically connect patients to methadone services [2]. Methadone has historically been classified as a “high‐barrier” option due to regulations that mandate daily dosing at opioid treatment programs (OTPs) [3]. Titration protocols are also gradual, causing many patients to experience withdrawal during the initiation period, which may further complicate linkage [4].

Despite these barriers, there is growing interest in developing expedited pathways to methadone treatment for ED and hospital patients [4, 5]. Accelerated protocols for methadone initiation during hospitalization have been proposed, and bridge clinics administering methadone under the “72‐h rule” have facilitated transitions between hospitals and traditional OTPs [5]. However, there is limited evidence on the feasibility or effectiveness of direct OTP referral following discharge from the ED setting [6, 7, 8, 9].

In this study, we evaluated the implementation of a clinical pathway to rapidly connect patients to an OTP following discharge from the ED or hospital. Our objective was to describe outcomes for patients referred to the pathway.

We conducted a retrospective cohort study. The study period was January 2023 to August 2024. The methadone pathway was implemented in three hospitals in an academic health system located in Philadelphia. Patients were referred to a partnering community OTP that provided expedited intake appointments and wrap‐around treatment services including methadone initiation and titration, peer counseling, behavioral health services, and social work. This study was approved by the University of Pennsylvania Institutional Review Board.

The pathway consisted of four key components: (1) identification of ED patients with OUD who elect methadone treatment, with optional methadone initiation in the ED or hospital, (2) expedited referral to the community OTP, (3) peer support and navigation services, and (4) ED bridge dosing of methadone during gaps in outpatient treatment access.

First, ED patients who were identified to have OUD were offered treatment options including buprenorphine, as previously described [10]. Patients who opted for outpatient methadone treatment received additional testing prompted by an electronic health record (EHR) order set, including electrocardiogram (ECG), urine drug screen (UDS), and urine pregnancy test. Those with Clinical Opiate Withdrawal Scale (COWS) greater than 8 were offered a single dose of methadone (30 or 40 mg) and adjunct treatment for opioid withdrawal. Patients who required medical admission remained eligible for the pathway after hospital discharge, as well as those who opted for brief inpatient (< 72 h) OUD treatment in an on‐site unit where they could receive methadone.

Second, patients were referred to an intake appointment at the partner OTP through the CareConnect Warmline, a telehealth substance use navigation and peer recovery specialist (PRS) service [11]. During operating hours (9a—9p, 7 days per week), peers engaged with patients, coordinated with the OTP, and scheduled a next‐day intake appointment. Referrals outside of regular hours were processed the next day.

Third, CareConnect staff engaged with patients during and after the ED encounter to offer support, address health‐related social needs, and help them navigate to treatment. Patients could receive transportation vouchers, assistance with shelter intake, and pre‐paid cell phones in addition to harm reduction counseling.

Fourth, the pathway facilitated bridge dosing of methadone in the ED until patients completed OTP intake, anticipating a lack of available appointments over the weekend or other unexpected delays. To facilitate this process, an on‐call emergency or addiction medicine physician documented treatment plans in the EHR, including dosing guidance for ED clinicians and pharmacists.

This study included patients who were referred to the pathway and engaged with CareConnect during the ED encounter. To examine the impact of this pathway on individuals not actively receiving methadone, we included patients with no prior experience with methadone or those who were resuming treatment after an extended gap (> 1 month). We excluded patients who were transferred to long‐term inpatient substance use disorder treatment from the ED or hospital.

We extracted patient demographic and clinical data from the EHR. Additional clinical information was extracted from a REDCap database that was completed as part of intake for CareConnect, including housing status and previous OUD treatment history. We used structured chart review of clinical documentation for the ED or hospital encounter to identify patient‐reported co‐use of substances and urine drug testing results.

The primary outcome was completion of an OTP intake appointment within 30 days of ED or hospital discharge. Secondary outcomes included engagement with OTP services at 30 days (including ongoing methadone administration and/or program attendance), time to intake, engagement with peer services following discharge, and ED bridge dosing. To determine these outcomes, patients were matched to OTP records using multiple identifiers. We used descriptive statistics to tabulate patient characteristics and outcomes, stratified by discharging hospital unit.

There were 42 total referrals to the outpatient methadone clinical pathway during the study period, including 13 (31%) patients discharged from the ED, 23 (55%) after hospital admission, and 6 (14%) after discharge from brief inpatient OUD treatment (Table 1). Two patients were referred to non‐partner OTP sites; outcomes for these individuals are not reported. For context, there were 1270 total ED or hospital encounters for OUD during the study period (Data S1).

**TABLE 1:** Outcomes and characteristics for patients referred to hospital methadone linkage pathway.

Patient outcomes	All referrals (*n* = 40)	ED referrals (*n* = 13)	Hospital referrals (*n* = 21)	Inpatient OUD treatment referrals (*n* = 6)
Completed intake at OTP within 30 days, *n* (%)	25 (63)	11 (85)	10 (48)	4 (67)
Engagement with OTP services at 30 days, *n* (%)	22 (55)	9 (69)	10 (48)	3 (50)
Days to OTP intake, mean (SD)	1.4 (1.3)	2.0 (1.4)	1.1 (1.2)	0.5 (0.6)
Engagement with hospital peer support following discharge, *n* (%)	17 (43)	10 (77)	6 (29)	1 (17)
Returned to ED for methadone bridge dose, *n* (%)	6 (15)	3 (23)	3 (14)	0 (0)

Patient characteristics	All referrals (*n* = 42)^a^	ED referrals (*n* = 13)	Hospital referrals (*n* = 23)	Inpatient OUD treatment referrals (*n* = 6)
Age, mean (SD)	43 (11)	43 (11)	45 (12)	38 (6)
Sex, *n* (%)				
Male	21 (50)	8 (62)	10 (43)	3 (50)
Female	21 (50)	5 (38)	13 (57)	3 (50)
Race and ethnicity				
Non‐Hispanic Black	12 (29)	4 (31)	6 (26)	2 (33)
Non‐Hispanic White	23 (55)	6 (46)	14 (61)	3 (50)
Hispanic	3 (7)	1 (8)	2 (9)	0 (0)
Other	4 (10)	2 (15)	1 (4)	1 (17)
Insurance				
Medicaid	37 (88)	10 (77)	22 (96)	5 (83)
Medicare	4 (10)	2 (15)	1 (4)	1 (17)
Commercial	1 (2)	1 (8)	0 (0)	0 (0)
Housing status				
Unhoused or unstable	11 (26)	2 (15)	5 (22)	4 (67)
Stable	31 (74)	11 (85)	18 (78)	2 (33)
Previous medication treatment for opioid use disorder				
Methadone	17 (40)	6 (46)	11 (48)	0 (0)
Buprenorphine	3 (7)	1 (8)	2 (9)	0 (0)
Both methadone and buprenorphine	21 (50)	6 (46)	10 (43)	5 (83)
None	1 (2)	0 (0)	0 (0)	1 (17)
Methadone dose administered prior to discharge, *n* (%)	39 (93)	10 (77)	23 (100)	6 (100)
Prior engagement with OTP in preceding 12 months	24 (57)	6 (46)	14 (61)	4 (67)
Co‐substance use^b^				
Cocaine	22 (52)	7 (54)	10 (43)	5 (83)
Amphetamine	8 (19)	1 (8)	5 (22)	2 (33)
Benzodiazepine	18 (43)	4 (31)	10 (43)	4 (67)
Alcohol	3 (7)	0 (0)	2 (9)	1 (17)
THC	8 (19)	1 (8)	4 (17)	3 (50)

Abbreviations: ED, emergency department; OTP, opioid treatment program; THC, tetrahydrocannabinol.

^a^ Includes additional patients referred to OTP services with whom there was no data sharing agreement for patient outcomes.

^b^ Determined from urine drug screen when available and/or ED chart review.

Of all patients referred to the pathway, 25 (63%) completed OTP intake within 30 days (Table 1). More than half of patients remained engaged with OTP services at 30 days (22, 55%), and the mean time between discharge and OTP intake was 1.4 days (SD 1.3). Nearly half of patients engaged with peer support services from CareConnect following discharge (17, 43%); and 6 (15%) used the ED at least once to obtain bridge methadone dosing. Of patients discharged from the ED, 11 (85%) completed OTP intake within 30 days and 9 (69%) remained engaged with the OTP at 30 days.

Few studies have described outcomes for methadone linkage pathways that are designed for ED patients (Data S1) [6, 9]. While there is growing evidence describing pathways for patients following hospital admission, these efforts have not been coordinated with the ED phase of care [7, 8]. Furthermore, many patients are keen to initiate outpatient treatment directly from the comunity, and may not require hospital admission or extended rehabilitation prior to beginning treatment [5]. The ED can serve as a bridge for patients to access expedited OTP linkage, which may be one explanation for the high intake completion and treatment retention rates in this study. Over 90% of the patients in this study had received methadone treatment in the past, suggesting that most patients were likely to be familiar with OTP requirements.

Our findings support the value of a low‐barrier ED and hospital methadone pathway in expanding options for patients to access MOUD. Merely the availability of the pathway offers a new opportunity for patients, in shared decision‐making with their providers, to expedite treatment initiation. This option is needed in the context of high fentanyl prevalence in the unregulated drug supply, leading some patients to decline buprenorphine or experience complications during initiation [3, 4]. While methadone is highly effective, access is often siloed from other parts of the healthcare system, creating considerable barriers for patients and clinicians. Of note, the pathway did not receive a high volume of referrals, accounting for 3% of patients with OUD encounters. It is possible that greater engagement by peers and clinical champions may further increase referrals, as could normalization of methadone as a viable OUD treatment option among ED clinicians.

When layered upon existing infrastructure for OUD treatment, development of the pathway involved few additional investments apart from minor EHR adaptations, clinician education, and a strong relationship with a partnering OTP. Our findings point to the importance of certain features, including access to peer services and ED bridge dosing. The pathway relied upon peer expertise to navigate patients through transitions between care settings and address social needs, including transportation and housing, to allow individuals the best chance at accessing treatment [4].

This study had limitations. First, this study was retrospective with the potential for missing enrollment or outcomes data. Second, this study was conducted within a single academic health system in an urban setting, limiting generalizability to other settings. Third, we were unable to conclude which patient characteristics or pathway components were associated with successful OTP linkage, although we present outcomes stratified by patient characteristics in the Data S1. Fourth, we did not assess abstinence from illicit opioid agonists as a study outcome.

In summary, nearly two‐thirds of patients who were referred to an outpatient methadone clinical pathway completed intake and remained engaged in treatment after 30 days. ED and hospital leaders may seek to develop linkage pathways specifically for outpatient methadone to augment existing OUD treatment strategies. Further evaluation is needed to determine the essential components of these pathways, identify strategies to increase adoption, and compare outcomes with referral to other modalities of OUD treatment.

## Disclosure

No large language model or other artificial intelligence tool was used to prepare this manuscript.

## Supporting information


**Data S1:** acem70135‐sup‐0001‐Supinfo1.docx.

## Data Availability

The data that support the findings of this study are available on request from the corresponding author. The data are not publicly available due to privacy or ethical restrictions.
