# Supplementary material for: Linkage to Outpatient Methadone Treatment From the Emergency Department and Hospital
Source: Acad Emerg Med. 2025 Aug 22;32(11):1251–4. doi: 10.1111/acem.70135 (PMC12608057; doi:10.1111/acem.70135)
Supplement: Supplementary file 1 — Data S1: acem70135‐sup‐0001‐Supinfo1.docx. [file ACEM-32-1251-s001.docx]

Linkage to outpatient methadone treatment from the emergency department and hospital

**Methods and Background Supplement**

1. **Table.** ED and hospital patients with opioid use disorder at study hospitals
2. **Table.** Characteristics of patients referred to hospital methadone linkage pathway, stratified by attainment of primary outcome (OTP intake within 30 days)
3. **Table.** Comparison of studies involving ED or hospital linkage pathways for outpatient methadone treatment

**Table.** ED and hospital patients with opioid use disorder at study hospitals

|  | **All Hospitals** | | **Hospital A** | | **Hospital B** | | **Hospital C** | |
| --- | --- | --- | --- | --- | --- | --- | --- | --- |
|  | **n** | **%** | **n** | **%** | **n** | **%** | **n** | **%** |
| Total visits for opioid use disorder | 1270 |  | 314 |  | 720 |  | 236 |  |
| Buprenorphine prescribed at discharge | 220 | 17% | 27 | 9% | 174 | 24% | 19 | 8% |
| Methadone administered during encounter | 406 | 32% | 136 | 43% | 223 | 31% | 47 | 20% |

|  | **All Units** | | **ED** | | **Observation** | | **Inpatient** | |
| --- | --- | --- | --- | --- | --- | --- | --- | --- |
|  | **n** | **%** | **n** | **%** | **n** | **%** | **n** | **%** |
| Total visits for opioid use disorder | 1270 |  | 873 |  | 81 |  | 316 |  |
| Buprenorphine prescribed at discharge | 220 | 17% | 158 | 18% | 54 | 67% | 8 | 3% |
| Methadone administered during encounter | 406 | 32% | 144 | 16% | 40 | 49% | 222 | 70% |

- Inclusion criteria
  - ICD-10 code for opioid-related visit (F11.1-2, T40.X)
  - Three hospital study sites
  - ED, observation, and inpatient encounters
  - 1/1/2023 – 8/30/2024
- Outcomes
  - Buprenorphine: prescription at time of ED, observation, inpatient discharge
  - Methadone: at least 1 dose of methadone ordered and administered during ED, observation, or inpatient encounter

**Table.** Characteristics of patients referred to hospital methadone linkage pathway, stratified by attainment of primary outcome (OTP intake within 30 days)

|  | | **n (%)** | | |
| --- | --- | --- | --- | --- |
| **Characteristic** | | **Primary Cohort**  **(n = 40)** | **Intake within 30 days**  **(n = 25)** | **No intake within 30 days (n = 15)** |
| Age, mean (SD) | | 43.1 (11.1) | 44.2 (11.5) | 41.3 (10.5) |
| Sex | |  |  |  |
|  | Male | 20 (50.0) | 14 (56.0) | 6 (40.0) |
|  | Female | 20 (50.0) | 11 (44.0) | 9 (60.0) |
| Race and ethnicity | |  |  |  |
|  | Black (non-Hispanic) | 12 (31.6) | 6 (24.0) | 6 (40.0) |
|  | White (non-Hispanic) | 22 (57.9) | 14 (56.0) | 8 (53.3) |
|  | Hispanic | 2 (5.0) | 1 (4.0) | 1 (6.7) |
|  | Other | 4 (10.0) | 4 (16.0) | 0 (0.0) |
| Insurance | |  |  |  |
|  | Medicaid | 36 (90.0) | 21 (84.0) | 15 (100.0) |
|  | Medicare | 3 (7.5) | 3 (12.0) | 0 (0.0) |
|  | Private | 1 (2.5) | 1 (4.0) | 0 (0.0) |
| Housing status | |  |  |  |
|  | Unhoused | 9 (22.5) | 3 (12.0) | 6 (40.0) |
|  | Unstable housing | 1 (2.5) | 1 (4.0) | 0 (0.0) |
|  | Permanent housing | 30 (75.0) | 21 (84.0) | 9 (60.0) |
| Previous medication treatment for opioid use disorder | |  |  |  |
|  | Methadone | 15 (37.5) | 12 (48.0) | 3 (20.0) |
|  | Buprenorphine | 3 (7.5) | 2 (8.0) | 1 (6.7) |
|  | Both | 21 (52.5) | 11 (44.0) | 10 (66.7) |
|  | None | 1 (2.5) | 0 (0.0) | 1 (6.7) |
| Setting of methadone initiation | |  |  |  |
|  | ED | 24 (60.0) | 15 (60.0) | 9 (60.0) |
|  | Hospital | 8 (20.0) | 4 (16.0) | 4 (26.7) |
|  | Detox | 5 (12.5) | 4 (16.0) | 1 (6.7) |
|  | Methadone not initiated | 3 (7.5) | 2 (8.0) | 1 (6.7) |
| Co-substance use | |  |  |  |
|  | Cocaine | 20 (50.0) | 10 (40.0) | 10 (66.7) |
|  | Amphetamine | 7 (17.5) | 3 (12.0) | 4 (26.7) |
|  | Benzodiazepine | 17 (42.5) | 10 (40.0) | 7 (46.7) |
|  | Barbiturate | 2 (5.0) | 2 (8.0) | 0 (0.0) |
|  | THC | 8 (20.0) | 5 (20.0) | 3 (20.0) |
|  | Alcohol | 3 (7.5) | 3 (12.0) | 0 (0.0) |
|  | PCP | 2 (5.0) | 2 (8.0) | 0 (0.0) |
|  | None | 9 (22.5) | 8 (32.0) | 1 (6.7) |
| Prior engagement with OTP in preceding 12 months | | 23 (57.5) | 16 (64.0) | 7 (46.7) |
| Returned to ED for methadone bridge dose | | 6 (15.0) | 4 (16.0) | 2 (13.3) |
| Engagement with hospital peer support following discharge | | 17 (42.5) | 14 (56.0) | 3 (20.0) |

**Table.** Comparison of studies involving ED or hospital linkage pathways for outpatient methadone treatment

|  | Wolfson *et al.* (2024) | Calcaterra *et al.* (2024) | Tierney *et al. (2023)* | Huo *et al.* (2025) | Kessler *et al.* (2022) | Taylor *et al.* (2022) | Skogrand *et al.* (2024) | Bowman *et al.* (2024) | This study |
| --- | --- | --- | --- | --- | --- | --- | --- | --- | --- |
| **Study design** | | | | | | | | | |
| Type | Open trial^1^ | Retrospective cohort study | Retrospective cohort study | Retrospective cohort study | Retrospective cohort study | Retrospective cohort study | Quality | Quality | Retrospective cohort study |
| Period | Feb 2023 – Sep 2023 | Apr 2019 –  Feb 2022 | Oct 2017 –  Jul 2019 | Jan 2021 –  Aug 2021 | Dec 2016 – Dec 2019 | Mar 2021 –  Aug 2021 | 2022 | 2022-2023 | Jan 2023 –  Aug 2024 |
| Size | 28 patients* | 73 patients (96 enrollments) | 125 patients | 62 patients* | 46 patients* | 113 patients (121 treatment episodes) | 37 patients | 36 patients | 42 patients |
| Population | ED patients | Inpatient admissions | Inpatient admissions | ED patients and inpatient admissions | Inpatient admissions | Bridge clinic patients | Inpatient admissions | ED patients and inpatient admissions | ED patients, inpatient admissions, short inpatient SUD treatment |
| Setting and location | 1 urban hospital  Burlington, VT | 1 urban hospital  Aurora, CO | 1 urban hospital  San Francisco, CA | 1 urban hospital  Camden, NJ | 1 urban hospital  St. Louis, MO | 1 urban hospital  Boston, MA | 1 urban hospital  Portland, OR | 2 urban hospitals  Baltimore, MD | 3 urban hospitals  Philadelphia, PA |
| **Outcomes** | | | | | | | | | |
| OTP intake completion rate | 12/28 (43%) | 56/73 (77%) | 50/125 (40%) | 41/62 (66%) | N/A | 105/121 (87%) | N/A | N/A | 25/40 (62.5%) |
| Treatment retention rate | N/A | N/A | 30-day:  37/125 (30%) | 30-day:  38/62 (61%) | 30-day:  18/46 (39%) | 30-day:  70/121 (58%) | N/A | N/A | 30-day:  22/40 (55%) |
| Days until intake | N/A | N/A | Median 1 (IQR 1-2) | N/A | N/A | 101 had intake within 2 days | N/A | N/A | Mean 1.4 (SD 1.3) |
| **Pathway components** | | | | | | | | | |
| Peer support | Yes | N/A | N/A | N/A | Yes | N/A | Yes** | Yes | Yes |
| Engagement after discharge | Yes | Yes  (only if intake not completed within 24 hr) | N/A | N/A | N/A | Yes | Yes | N/A | Yes |
| Bridge dosing | Yes  Required daily return to ED but without check-in (doses held in lockbox) | Yes  Up to 3-day take-home supply dispense | N/A | Yes  Required daily return | N/A | Yes  Required daily return | Yes  Up to 3-day take-home supply dispense | Yes  Up to 3-day take-home supply dispensed | Yes  Required daily return to ED |
| *These *n* only includes patients who were initiated on methadone in the ED/hospital with intention to link to OTP. Not included in this table but included in the respective studies: additional patients who were already established in care at an OTP, were not referred to partnering OTPs, were referred to buprenorphine treatment, or were admitted to the hospital. | | | | | | | | | |
| Inclusion criteria for studies included in this comparison:   - N > 3 patients - Describes pathway from hospital to outpatient methadone without intervening residential or inpatient substance use disorder treatment - Published after 2020 | | | | | | | | | |

**References**

1. Bowman LA, Berger O, Nesbit S, Stoller KB, Buresh M, Stewart R. Operationalizing the new DEA exception: A novel process for dispensing of methadone for opioid use disorder at discharge from acute care settings. Am J Health Syst Pharm 2024;81:204-18.
2. Calcaterra SL, Saunders S, Grimm E, et al. In-Hospital Methadone Enrollment: a Novel Program to Facilitate Linkage from the Hospital to the Opioid Treatment Program for Vulnerable Patients with Opioid Use Disorder. Journal of General Internal Medicine 2024;39:385-92.
3. Huo S, Heil J, Salzman MS, et al. Emergency department utilization of the methadone “72-hour rule” to bridge or initiate and link to outpatient treatment. The American Journal of Emergency Medicine 2025;89:209-15.
4. Kessler SH, Schwarz ES, Liss DB. Methadone vs. Buprenorphine for In-Hospital Initiation: Which Is Better for Outpatient Care Retention in Patients with Opioid Use Disorder? Journal of Medical Toxicology 2022;18:11-8.
5. Skogrand E, Sharpe J, Englander H. Dispensing Methadone at Hospital Discharge: One Hospital's Approach to Implementing the "72-hour Rule" Change. J Addict Med 2024;18:71-4.
6. Taylor JL, Laks J, Christine PJ, et al. Bridge clinic implementation of “72-hour rule” methadone for opioid withdrawal management: Impact on opioid treatment program linkage and retention in care. Drug and Alcohol Dependence 2022;236:109497.
7. Tierney HR, Takimoto SW, Azari S, Steiger S, Martin M. Predictors of Linkage to an Opioid Treatment Program and Methadone Treatment Retention following Hospital Discharge in a Safety-Net Setting. Subst Use Misuse 2023;58:1172-6.
8. Wolfson D, King R, Lamberson M, et al. Methadone Initiation in the Emergency Department for Opioid Use Disorder. West J Emerg Med 2024;25:668-74.
